# Depression, anxiety levels and sleep quality indexes among the spouses of people with epilepsy

**DOI:** 10.1590/0004-282X-ANP-2020-0207

**Published:** 2021-05-09

**Authors:** Halil ONDER, Ersin Kasim ULUSOY, Caner BAYDAR, Mustafa KIRAZ, Muhammet Okay ORUN, Zehra KILIÇARSLAN, Merve BASOL, Aygul TANTIK

**Affiliations:** 1 Yozgat City Hospital, Department of Neurology, Yozgat, Turkey. Yozgat City Hospital Department of Neurology Yozgat Turkey; 2 Kayseri City Hospital, Department of Neurology, Kayseri, Turkey. Kayseri City Hospital Department of Neurology Kayseri Turkey; 3 Van Training and Research Hospital, Department of Neurology, Van, Turkey. Van Training and Research Hospital Department of Neurology Van Turkey; 4 Yozgat City Hospital, Department of Psychology, Yozgat, Turkey. Yozgat City Hospital Department of Psychology Yozgat Turkey; 5 Hacettepe University Medical School, Department of Biostatistics, Ankara, Turkey. Hacettepe University Hacettepe University Medical School Department of Biostatistics Ankara Turkey; 6 Taksim Training and Research Hospital, Department of Neurology, Istanbul, Turkey. Taksim Training and Research Hospital Department of Neurology Istanbul Turkey

**Keywords:** Epilepsy, Sleep, Seizures, Anxiety, Depression, Epilepsia, Sono, Convulsões, Ansiedade, Depressão

## Abstract

**Background:**

Psychiatric problems and sleep disturbances are comorbidities that are frequently encountered among people with epilepsy. However, their presence among the spouses of peoples with epilepsy remains to be elucidated.

**Objective:**

The objective of this study was to evaluate the spouses of people with epilepsy (PWE), with and without a history of seizures during sleep, in terms of depression, anxiety and sleep quality.

**Methods:**

This prospective, cross-sectional study was conducted in three groups of 18 to 65-year-olds. Group 1 consisted of healthy spouses of 127 healthy volunteers without any known neurological disease; group 2 comprised spouses of 63 PWE who had no history of seizure during sleep; and group 3 consisted of spouses of 63 PWE who had a history of at least one seizure during sleep in the course of the previous year. Questionnaires seeking demographic data and the Pittsburgh Sleep Quality Index (PSQI), Beck Depression Inventory and Beck Anxiety Inventory were applied to all participants.

**Results:**

The depression scores of the group of spouses of PWE were higher than those of the control group and were higher in group 3 than in group 2 (p = 0.017). The anxiety scores of the group of spouses of PWE were significantly higher than those of the control group, but no difference in anxiety scores was found between group 2 and group 3 (p = 0.170). The mean PSQI score of group 3 was higher than that of group 2 (p = 0.029). However, regression analyses did not show any difference between these groups.

**Conclusion:**

We found that the PSQI scores, which reflected sleep quality, were higher among the spouses of PWE who had seizures during sleep and who had more severe epilepsy.

## RESUMO

**Introdução:**

Problemas psiquiátricos e distúrbios do sono são comorbidades muito encontradas em pessoas com epilepsia. No entanto, sua presença nos cônjuges de pessoas com epilepsia ainda precisa ser elucidada.

**Objetivo:**

O objetivo do presente estudo foi avaliar os cônjuges de pessoas com epilepsia (PCE) com e sem histórico de convulsões durante o sono em termos de depressão, ansiedade e qualidade do sono.

**Métodos:**

Este estudo transversal prospectivo foi realizado com três grupos de jovens de 18 a 65 anos. O Grupo 1 era formado por cônjuges saudáveis ​​de 127 voluntários saudáveis ​​sem uma doença neurológica conhecida; o grupo 2 era composto por cônjuges de 63 PCE que não tinham histórico de convulsões durante o sono; o grupo 3 consistiu em cônjuges de 63 PCE que tinham história de pelo menos uma convulsão durante o sono no ano anterior. O questionário incluindo dados demográficos, Índice de Qualidade do Sono de Pittsburgh (PSQI), Inventário de Beck para Depressão e Inventário de Beck para Ansiedade foi aplicado a todos os participantes.

**Resultados:**

Os escores de depressão do grupo de cônjuges de PCE foram maiores que os do grupo controle e foram maiores no grupo 3 em relação ao grupo 2 (p=0,017). Os escores de ansiedade do grupo de cônjuges de PCE foram significativamente maiores do que os do grupo controle. No entanto, não foi encontrada diferença nos escores de ansiedade entre o grupo 2 e o grupo 3 (p=0,170). Os escores médios do PSQI do grupo 3 foram maiores do que a pontuação média do grupo 2 (p=0,029). Apesar disso, as análises de regressão não revelaram nenhuma diferença entre esses grupos.

**Conclusão:**

Verificamos que os escores do PSQI que refletem a qualidade do sono foram maiores nos cônjuges de PCE, que tiveram convulsões durante o sono e que apresentaram epilepsia mais grave.

## INTRODUCTION

Epilepsy is a common and chronic neurological disease that is characterized by recurrent seizures. These impose a major burden on patients, their caregivers and society. Although epilepsy is a neurological disease, it unarguably results in a variety of physical and psychosocial consequences. Moreover, previous studies have demonstrated that the caregivers of people with epilepsy (PWE) also face many disease-related restrictions. The risk of social and psychological burdens among caregivers (who are generally their spouses) has also been found to be high^1^^,^^2^^,^^3^^,^^4^. It has also been reported that these caregivers have poor quality of life, just like PWE^4^^,^^5^. Higher levels of depression and anxiety have been found among the caregivers of PWE, in comparison with the control group, in several recent reports^6^^,^^7^^,^^8^. However, although studies on sleep quality indexes among PWE have already been conducted^9^^,^^10^^,^^11^^,^^12^, research addressing the sleep quality of the caregivers of PWE is extremely rare^13^^,^^14^.

In a single report by Hamamci et al.^13^, the spouses of PWE were specifically investigated in terms of anxiety, depression and sleep quality. These authors concluded that sleep seizures had a negative effect on the scores of indexes that reflect the anxiety, depression, and sleep quality of the spouses of PWE. However, in that study, no validated evaluation of the severity of seizures was conducted between groups of spouses of patients with and without seizures during sleep.

We hypothesized that the severity of seizures would possibly differ between these two groups. Moreover, this could be a crucial factor that might be efficient for investigating the association between sleep quality of the spouses of PWE and the presence of sleep seizures.

In the light of these discussions, we aimed in this study to investigate the spouses of PWE with and without seizures during sleep, in terms of depression, anxiety and sleep quality, in a larger group of patients. In particular, we also aimed to investigate the impact of the severity of epilepsy on these associations and its possible role as a confounding variable for the relationship between seizures during sleep and the parameters of depression, anxiety and sleep quality.

## METHODS

This was a cross-sectional case-control study. The individuals who agreed to participate in the study were informed about it and they all signed an informed consent statement. This study was approved by the local ethics committee of Yozgat City Hospital.

### Study population

PWE who had been married for at least one year and whose diagnoses of epilepsy were in accordance with the criteria of the International League Against Epilepsy were enrolled in this study^15^. The study group consisted of patients who were being followed up at four neurology clinics in different centers in Turkey between April 2019 and December 2019. All of the patients had had their diagnoses of epilepsy for more than one year. These patients and their spouses were included in the study. Furthermore, healthy volunteers and their healthy spouses, who had been married for at least one year and had been admitted to these neurology clinics with nonspecific symptoms like tinnitus, dizziness or mild headache formed the control group. Individuals with a diagnosis of neurological disease or any chronic disease were not included in the study.

A total of 63 patients without a history of seizures during sleep (group 2) and 63 patients with at least one seizure during sleep (group 3) were enrolled in the study together with their spouses who were 18-65 years of age and could understand the research and fill out the questionnaires. We evaluated the presence of seizures during sleep according to the results from the interviews with the patients and their spouses.

We asked the couples whether they regularly shared the same bed or not, and we excluded couples who were not sharing the same bed or were not in the same bed during the seizure. The spouses of PWE who had alcohol, substance or caffeine addiction, chronic physical disease, a diagnosis of epilepsy, a history of neurological diseases that affected cognitive skills, any physical or psychiatric illness that caused sleep disturbance or regular use of sleeping pills (more than two sleeping pills used per month) were excluded from the study. In addition, patients or their spouses who were pregnant or lactating, or were working in a job that involved a night shift, were excluded from the study.

The Pittsburgh Sleep Quality Index (PSQI), the Beck Depression Inventory (BDI) and the Beck Anxiety Inventory (BAI) were administered to the spouses of PWE and to the controls. In addition, the National Hospital Seizure Severity Scale was applied to the epilepsy patients. For a more detailed investigation between epilepsy during sleep and sleep quality among the patients’ spouses, grading to evaluate the frequency of sleep seizures during the last one year was applied to all the spouses of PWE (grade 1: the patient had one seizure over the last 12 months; grade 2: 2-4 seizures over the last 12 months; and grade 3: more than 4 seizures over the last 12 months).

The control group (group 1) comprised 127 healthy volunteers who were similar to the study group in terms of marriage duration. All the exclusion criteria mentioned above were also valid for the control group. If one of the spouses had a history of seizures, the couple was excluded from the study.

#### Data collection questionnaire

A data collection questionnaire was prepared by the researchers for the purposes of this study. It asked for general information about the participants and was applied at the first contact with the patients. The following data were sought: age, gender, marital status, total education duration, anti-epileptic drug (AED) usage, detailed history of seizures including seizure type, frequency, duration, etc. and history of concomitant diseases.

#### Beck Depression Inventory

The BDI was developed by Beck et al.^16^ to evaluate the physical, emotional and cognitive symptoms and motivation that are seen in depression. The validity and reliability study on the BDI was performed by Hisli^17^.

#### Beck Anxiety Inventory

The BAI was developed by Beck et al.^18^ in order to assess the extent of anxiety symptoms in individuals. The validity and reliability study on the BAI was performed by Ulusoy et al.^19^.

#### Pittsburgh Sleep Quality Index

The PSQI was developed by Buysse et al.^20^, and the Turkish validity and reliability study was carried out by Ağargün et al.^21^. The 18 questions scored on the scale form seven component subscales. These comprise subjective sleep quality, sleep latency, sleep duration, sleep efficiency, sleep disturbance, use of sleep medications and daytime dysfunction. A total PSQI score of 5 or more points indicates poor sleep quality.

#### The National Hospital Seizure Severity Scale

The National Hospital Seizure Severity Scale (NHS3) is a refined version of the Chalfont Seizure Severity Scale. It has been suggested that the NHS3 provides a valid, easily applicable measurement of seizure severity that is acceptably reliable for use in clinical trials^22^.

### Statistical analysis

The statistical analysis was performed using the SPSS 22.0 software (Statistical Package for the Social Sciences; IBM Inc., Chicago, IL, USA). Descriptive statistics on the data were calculated and the Kolmogorov-Smirnov and Shapiro-Wilk tests were applied to test the normality of distribution. The chi-square test was used for comparisons between groups regarding the categorical variables. The Mann-Whitney U test was used for two-group comparisons of continuous variables that did not fit normal distribution, and the analysis of variance (ANOVA) (Kruskal-Wallis H) test was used in comparisons between more than two groups. Pearson's correlation test was used for normally distributed data, and Spearman's correlation test was used for data not showing normal distribution. A regression model was established for the variables showing significant correlations, and diagnostic tests on this model were carried out. p < 0.05 was considered statistically significant.

## RESULTS

### Comparative analyses on the groups in terms of demographic properties

The demographic characteristics of the spouses of 126 people with epilepsy and 127 subjects in the control group are demonstrated in Table 1. The demographic characteristics of the 63 spouses of PWE with seizures during sleep and the 63 spouses of PWE without seizures are summarized in Table 2.

**Table 1.** Comparison of the demographic and clinical variables between the healthy volunteers and spouses of people with epilepsy**.**

		Controls (n = 127)	Spouses of people with epilepsy (n = 126)	p-value
Age (mean ± SD)		35.71 ± 10.55	32.71 ± 11.66	0.033*
Gender	Female [n (%)] Male [n (%)]	100 (78.7) 27 (21.3)	71 (56.3) 55 (43.7)	< 0.001*
Education status	Without education Primary school graduates Secondary school graduates High school graduates University graduates and postgraduates	1 (0.8) 31 (24.4) 15 (11.8) 25 (19.7) 55 (43.3)	6 (4.8) 31 (24.6) 33 (26.2) 38 (30.2) 18 (14.3)	< 0.001*
Education duration (years) (mean ± SD)		10.80 ± 4.23	8.78 ± 3.80	< 0.001*
BDI (mean ± SD)		11.36 ± 8.12	16.63 ± 10.57	< 0.001*
BDI classifications	None [n (%)] Mild [n (%)] Moderate [n (%)] Severe [n (%)]	63 (49.6) 37 (29.1) 23 (18.1) 4 (3.1)	40 (31.7) 28 (22.2) 43 (34.1) 15 (11.9)	< 0.001*
BAI		11.74 ± 10.04	17.40 ± 12.98	0.002*
BAI classifications	None [n (%)] Mild [n (%)] Moderate [n (%)] Severe [n (%)]	49 (38.6) 43 (33.9) 26 (20.5) 9 (7.1)	29 (23.0) 42 (33.3) 29 (23.0) 26 (20.6)	0.004*
PSQI (mean ± SD)		11.36 ± 4.37	9.16 ± 6.99	0.003*

*Statistically significant; SD: standard deviation; BDI: Beck Depression Inventory; BAI: Beck Anxiety Inventory; PSQI: Pittsburgh Sleep Quality Index.

**Table 2.** Comparisons of the demographic and clinical characteristics of the spouses of epilepsy patients with seizures during sleep and without seizures during sleep.

		Seizure during sleep		p-value
		Absent (n = 63)	Present (n = 63)	
Age [median (range)]		26 [17-58]	35 [18-65]	0.002*
Gender	Female [n (%)] Male [n (%)]	44 (69.8) 19 (30.2)	27 (42.9) 36 (57.1)	0.002*
Education status	Without education [n (%)] Primary school graduates [n (%)] Secondary school graduates [n (%)] High school graduates [n (%)] University graduates and postgraduates [n (%)]	3 (4.8) 18 (28.6) 11 (17.5) 21 (33.3) 10 (15.9)	3 (4.8) 13 (20.6) 22 (34.9) 17 (27.0) 8 (12.7)	0.268
Education status (years) [median (range)]		8 [0-15]	8 [0-15]	0.784
Marriage duration [median (range)]		1 [0-39]	7 [0-43]	0.012*
BDI [median (range)]		11 [2-43]	18 [0-55]	0.017*
BDI classification	None [n (%)] Mild [n (%)] Moderate [n (%)] Severe [n (%)]	26 (41.3) 16 (25.4) 14 (22.2) 7 (11.1)	14 (22.2) 12 (19.0) 29 (46.0) 8 (12.7)	0.024*
BAI [median (range)]		11 [2-62]	16 [1-55]	0.170
BAI classification	None [n (%)] Mild [n (%)] Moderate [n (%)] Severe [n (%)]	16 (25.4) 25 (39.7) 11 (17.5) 11 (17.5)	13 (20.6) 17 (27.0) 18 (28.6) 15 (23.8)	0.247
PSQI total score [median (range)]		6 [1-40]	9 [2-38]	0.029*
Bilateral tonic clonic seizure frequency	1-2 per year 3-6 per year 7-12 per year More than one per month	35 (55.6) 24 (38.1) 3 (4.8) 1 (1.6)	13 (20.6) 28 (44.4) 9 (14.3) 13 (20.6)	< 0.001*
GTCS	Absent [n (%)] Present [n (%)]	6 (9.5) 57 (90.5)	8 (12.7) 55 (87.3)	0.571
Multiple drug usage	Absent [n (%)] Present [n (%)]	52 (82.5) 11 (17.5)	33 (52.4) 30 (47.6)	< 0.001*
Number of AEDs [n (%)]	0 1 2 3 or more	1 (1.6) 50 (79.4) 12 (19.0) 9 (0.0)	0 (0.0) 33 (52.4) 20 (31.7) 10 (15.9)	< 0.001*
Change in seizure frequency after marriage	Absent [n (%)] Present [n (%)]	50 (79.4) 13 (20.6)	33 (52.4) 30 (47.6)	0.001*
Epilepsy duration [median (range)]		9 [1-41]	12 [1-45]	0.029*
NHS3 bilateral tonic clonic seizure subscore		12.92 ± 4.84	15.19 ± 5.00	0.011*
NHS3 total score [mean ± SD]		15.41 ± 8.50	21.28 ± 12.12	0.002*

*Statistically significant; SD: standard deviation; BDI: Beck Depression Inventory; BAI: Beck Anxiety Inventory; PSQI: Pittsburgh Sleep Quality Index; GTCS: generalized tonic clonic seizures; AEDs: anti-epileptic drugs; NHS3: National Hospital Seizure Severity Scale.

The comparative analyses between the control group (group 1) and the group of spouses of PWE revealed that the mean age and length of education were greater in the control group (p = 0.033 and p < 0.001, respectively). In addition, female gender was more prevalent in the control group (p <0.001). All of these constituted potential confounding factors.

The results from the comparative analyses on the demographic features between group 2 and group 3 are illustrated in Table 2.

### Analysis on Beck Depression Inventory and Beck Anxiety Inventory scores between groups

Regarding the BDI and BAI scores, higher-than-normal results were observed respectively in 68.3% and 77% of the spouses of PWE (Table 1). Comparisons between the control group (group 1) and all spouses of PWE (group 2 + group 3) revealed that the mean BDI score and BAI score were higher among the spouses of PWE (p ≤ 0.001 and p = 0.002 respectively). Comparisons between group 2 and group 3 revealed that the BDI scores were higher in group 3 (p = 0.017), whereas no difference in BAI scores was found between the groups (p = 0.170).

Further analyses after adjustments for the variables of age, gender and general seizure frequency did not reveal any significant difference in mean BDI scores between group 2 and group 3 (Table 3).

**Table 3.** Results from multiple regression analyses comparing the clinical variables of Beck Depression Inventory, Beck Anxiety Inventory and Pittsburgh Sleep Quality Index scores between the spouses of people with epilepsy with and without seizure during sleep, after adjusting for the independent variables of age, gender, National Hospital Seizure Severity Scale generalized tonic clonic seizure subscore and general seizure frequency. No significant differences were observed in relation to any of the variables.

	Model 1		Model 2 (adjusted)	
	b (b 95% CI)	p-value	b (b 95% CI)	p-value
BDI	0.166 (-0.096; 0.428)	0.212	0.042 (-0.251; 0.335)	0.777
BAI	0.130 (-0.137; 0.397)	0.338	-0.004 (-0.303; 0.295)	0.979
PSQI	0.224 (0.003; 0.444)	0.047	0.198 (-0.060; 0.455)	0.131

Model 1: independent variable group; Model 2: adjusted for the variables of age, gender, National Hospital Seizure Severity Scale generalized tonic clonic seizure subscore and seizure frequency. BDI: Beck Depression Inventory; BAI: Beck Anxiety Inventory; PSQI: Pittsburgh Sleep Quality Index; b: beta coefficient; p: probability of obtaining test results at least as extreme as the results actually observed, under the assumption that the null hypothesis is correct.

### Results from comparative analyses on Pittsburgh Sleep Quality Index scores between groups

Regarding the PSQI scores, 87% of group 1 (n = 111), 68% of group 2 (n = 43), and 75% of group 3 had scores reflecting poor sleep quality. Comparisons between control groups and spouses of epilepsy patients (group 2 + group 3) revealed that the PSQI scores were unexpectedly higher in the control group (p = 0.003). On the other hand, the PSQI scores of group 3 were higher than those of group 2, which showed that spouses of PWE with seizures during sleep had poorer sleep quality than spouses of those without seizures during sleep (p = 0.029). However, after adjustment for the variables of age, gender and general seizure frequency, there was no difference in PSWI scores between the groups (p = 0.131) (Table 1 and Table 2).

### Analyses on scores reflecting the severity of epilepsy between group 2 and group 3

Comparisons between group 2 and group 3 showed that the measurements had higher values in group 3, which reflected the severity of the seizures (Table 2).

### Results from the correlation analyses

There were weak positive correlations between the PSQI total score and the BDI and BAI scores. It was noted that the correlations between seizure severity scores during sleep and clinical variables, including BDI, BAI and PSQI, did not reveal any significant results. On the other hand, to understand the associations between the severity of epilepsy and sleep quality, some parameters such as the NHS3 total score and epilepsy duration were also included in the correlation analyses. These revealed a weak positive correlation between the NHS3 total score and PSQI scores (r = 0.205; p = 0.021). Lastly, in accordance with the expectations, there was a positive correlation between the NHS3 total score and the score for seizure frequency during sleep (Table 4).

**Table 4.** Results from correlation analyses.

		BDI		BAI		PSQI total	
		r	p-value	r	p-value	r	p-value
A)	Epilepsy duration	0.080	0.375	0.088	0.328	0.028	0.760
	Seizure frequency during sleep	0.170	0.057	0.094	0.296	0.013	0.884
	NHS3 total score	0.142	0.112	0.095	0.292	0.205	0.021*
	PSQI total score	0.331	0.000*	0.351	0.000*	-	-
B)	NHS3 total score	r	p				
	Seizure frequency during sleep	0.208	0.019*				

*Statistically significant; BDI: Beck Depression Inventory; BAI: Beck Anxiety Inventory; PSQI: Pittsburgh Sleep Quality Index; EDs: NHS3: National Hospital Seizure Severity Scale; r: coefficient of correlation.

### Effects of Beck Depression Inventory and Beck Anxiety Inventory variables on Pittsburgh Sleep Quality Index

Multiple regression analysis was performed to explain the effects of the BDI and BAI on the PSQI. The effects of the BDI and BAI on the PSQI were statistically significant (F = 19.917; p = 0.000), and these effects are presented in detail in Table 5.

**Table 5.** Effects of Beck depression scale and Beck anxiety scale variables on the Pittsburgh Sleep Quality Index score.

Model		Unstandardized coefficients		Standardized coefficients	**Student’s *t* test**	Sig.
		βa	Std. error	βb		
1	(Constant)	7.156	0.616		11.620	0.000*
	BDS	0.102	0.050	0.168	2.047	0.042*
	BAS	0.116	0.041	0.233	2.845	0.005*

*Statistically significant; a: dependent variable; PSQI: Pittsburgh Sleep Quality Index score; F = 19.917; p = 0.000; R^2^ = 0.137; βa: non-standardized regression coefficient; βb: standardized regression coefficient. BDS: Beck depression scale; BAS: Beck anxiety scale.

## DISCUSSION

In this study, we found that the BDI and BAI scores, which reflect the levels of depression and anxiety, were higher in the spouses of PWE than in the control group. The prevalence of anxiety and depressive disorders among PWE has been reported to be between 20 and 50%^23^^,^^24^^,^^25^. In the literature, depression and anxiety have also been reported to be more common among the spouses of PWE, in relation to the control group^4^^,^^13^^,^^26^. In the study by Hamamci et al., the BDI and BAI rates were 50 and 66.7%^13^. We found that BDI and BAI scores were notably higher than normal among the spouses of PWE, at rates of 68.3 and 77%, respectively. Higher burden levels, affliction due to stigma and increased depression and anxiety levels among the caregivers of PWE have often been reported previously, although these reports generally focused on the pediatric patient population^27^^,^^28^^,^^29^. However, the psychological status and sleep quality of the spouses of PWE, who are generally the caregivers of adult patients, have rarely been investigated^4^^,^^13^^,^^30^^,^^31^.

In the light of the data in the literature, the high rates of depression and anxiety indexes among the spouses of PWE in our study highlight the importance of evaluating psychiatric disturbances among these spouses. This matter continues to be underestimated within clinical practice.

The crucial point of the present study was that we also compared the presence of these comorbidities between spouses of PWE with and without seizures during sleep, which had only been investigated in a single study previously^13^. We found higher BDI scores among the spouses of PWE with seizures during sleep, as was reported in the previous study^13^, but we did not find any differences in BAI scores between our groups. On the other hand, we found that the scores for indexes that measure the severity of epilepsy, including the general seizure frequency, AED number, multiple AED usage rates, the NHS3 total score and NHSE bilateral tonic clonic seizure subscore, were all higher among the spouses of PWE with seizures during sleep. Moreover, we found positive correlations between the NHS3 total score and PSQI scores as well as between the NHS3 total score and the score for seizure frequency during sleep.

All these results highlight that seizure severity constitutes a crucial bias that needs to be kept in mind, in making comparisons between groups with seizures during sleep and without seizures. We noted that, after adjusting for the independent variables measuring seizure severity, there was no difference between the groups. This rather diminished the possible effect of sleep seizures as a factor determining the psychological status of the spouses of PWE.

These results may suggest that the severity of epilepsy may be a more significant determinant for use in predicting the depression and anxiety levels of the spouses of PWE. The severity of epilepsy is known to be associated with higher depression and anxiety levels in patients with epilepsy^32^^,^^33^^,^^34^. However, the impact of the clinical features of seizures on the spouses of these patients has not been widely investigated. In the crucial report by Zhu et al.^4^, seizure severity was found to be an independent predictor for worry relating to seizures and for impaired social functioning, among the caregivers of adult PWE. In another study, the burden imposed on caregivers, including depression, anxiety and stress scales, was found to be positively correlated with the patients’ seizure frequency per year^8^. However, no validated measurement regarding the severity of epilepsy was made in that study^8^. In another two reports^6^^,^^7^, increased seizure frequencies were found to be associated with higher levels of caregiving burden; however, the authors investigated caregivers overall, and did not specify the spouses of PWE as caregivers.

In this context, we also think that the results from our study may also provide substantial perspectives regarding the topic of marriage among people with epilepsy. It should be noted that although Hamamci et al. evaluated the annual number of seizures among spouses of PWE both with and without seizures during sleep, no validated questionnaire measuring the severity of epilepsy was administered^13^. Furthermore, those authors did not use these measurements (annual number of seizures) in regression analyses or correlation analyses for evaluating additional associations. This may have been the main limitation of their study. We think that the results from our study, which was conducted using methodology that was a highly similar to what Hamamci et al. used, provides valuable and also complementary data in this regard, given that our study also sought answers in relation to the limitations of their previous report.

On the other hand, contrary to expectations in our study, the PSQI scores were higher in the control group than among the spouses of PWE. We think that this may have been related to the heterogeneous nature of the study groups, in terms of age, gender and education status. On the other hand, the PSQI scores were higher among the spouses of PWE with seizures during sleep, which was also the case in the study by Hamamci et al.^13^. Nevertheless, it needs to be stated that, after adjusting for variables reflecting the seizure severity, no significant difference was found between the groups in terms of the PSQI scores. Moreover, no correlation was found between the PSQI scores and seizure frequency during sleep. However, significant correlations were found between the PSQI scores and the NHS3 total scores, which supports the idea that seizure severity may be a more valuable factor in determining the sleep quality of the spouses of PWE. Previous reports showed that the severity of epilepsy was associated with worse sleep quality among PWE^9^^,^^34^. However, to the best of our knowledge, the association between the severity of epilepsy and sleep indexes among the spouses of these patients had not been addressed previously.

The main limitation of our study was that our groups were not matched in terms of age, gender and education status, which may potentially have constituted confounding factors. It should be noted that, at the time of recruiting the control group, we invited many individuals of similar education levels to participate in our study. However, a high proportion of them refused to participate. In the end, the healthy control group was formed by subjects of rather higher educational level who had agree to participate in this study. We tried to overcome this limitation by repeating the comparisons after adjusting for these variables.

Another limitation may have arisen because the data regarding the specific AEDs were not investigated in detail. Psychiatric adverse events may vary according to the AEDs used: this is a known entity^35^ and potentially constitutes a crucial confounding factor in studies investigating the association between seizures and psychosocial status as well as sleep quality. Moreover, the PSQI subdomain scores were not evaluated in our study, which may have led to failure to reveal the specific features of sleep quality.

Lastly, although we have graded the sleep seizures in order to investigate the associations in detail and to use them in correlation analyses, these gradings (done according to their frequencies) had not previously been used and therefore lacked validation. Constituting a validated scale for sleep seizures in future related studies may provide substantial contributions in this regard.

## CONCLUSION

We found that indexes measuring depression, poor sleep quality and severity of epilepsy had higher scores among the spouses of PWE with seizures during sleep than among the spouses of PWE without seizures during sleep. However, after adjusting for the variables, including general seizure frequency, no differences between the groups were found in relation to any of the parameters. On the other hand, the results from the statistical analyses including correlation analyses suggested that seizure severity was considerably efficient as a factor interplaying with the depression, anxiety and sleep quality indexes of the spouses of PWE. We suggest that clinicians should remain attentive to the psychiatric health and sleep quality of the spouses of PWE, particularly those of the subgroup of patients suffering from more severe epilepsy. Through regarding the caregivers as a ‘co-target’ in this subgroup of patients, better quality of public health may be developed. This may also provide a multimodal therapeutic option for these subgroups of patients with more severe epilepsy. Confirmation of the results from our study through future reports on larger groups of cases is warranted, given that this may provide crucial perspectives for clinical care.
